# Seismic Hazards Implications of Uplifted Pleistocene Coral Terraces in the Gulf of Aqaba

**DOI:** 10.1038/s41598-017-00074-2

**Published:** 2017-02-24

**Authors:** W. Bosworth, P. Montagna, E. Pons-Branchu, N. Rasul, M. Taviani

**Affiliations:** 1 Apache Egypt Companies, 11 Street 281, New Maadi, Cairo Egypt; 2 Istituto di Scienze Marine (ISMAR), CNR, Via Gobetti 101, 40129 Bologna, Italy; 3 Laboratoire des Sciences du Climat et de l’Environnement LSCE/IPSL, CEA-CNRS-UVSQ, Université Paris-Saclay, Avenue de la Terrasse, Gif-sur-Yvette, Île-de-France 91198 France; 4 Lamont-Doherty Earth Observatory, Columbia University, 61 Route 9W, Palisades, 10964 NY USA; 5 Saudi Geological Survey, Box 54141, Jeddah, 21514 Saudi Arabia; 6 Biology Department, Woods Hole Oceanographic Institution, 266 Woods Hole Road, Woods Hole, 02543 MA USA; 7 Stazione Zoologica Anton Dohrn, Villa Comunale, 80121 Naples Italy

## Abstract

The Gulf of Aqaba transform plate boundary is a source of destructive teleseismic earthquakes. Seismicity is concentrated in the central sub-basin and decreases to both the north and south. Although principally a strike-slip plate boundary, the faulted margins of the Gulf display largely dip-slip extensional movement and accompanying footwall uplift. We have constrained rates of this uplift by measurements of elevated Pleistocene coral terraces. In particular the terrace that formed during the last interglacial (~125 ka) is found discontinuously along the length of the Gulf at elevations of 3 to 26 m. Global sea level was ~7 m higher than today at 125 ka indicating net maximum tectonic uplift of ~19 m with an average rate of ~0.015 cm/yr. Uplift has been greatest adjacent to the central sub-basin and like the seismicity decreases to the north and south. We suggest that the present pattern of a seismically active central region linked to more aseismic areas in the north and south has therefore persisted for at least the past 125 kyr. Consequently the potential for future destructive earthquakes in the central Gulf is greater than in the sub-basins to the north and south.

## Introduction

### Geologic Setting

**Figure 1:**
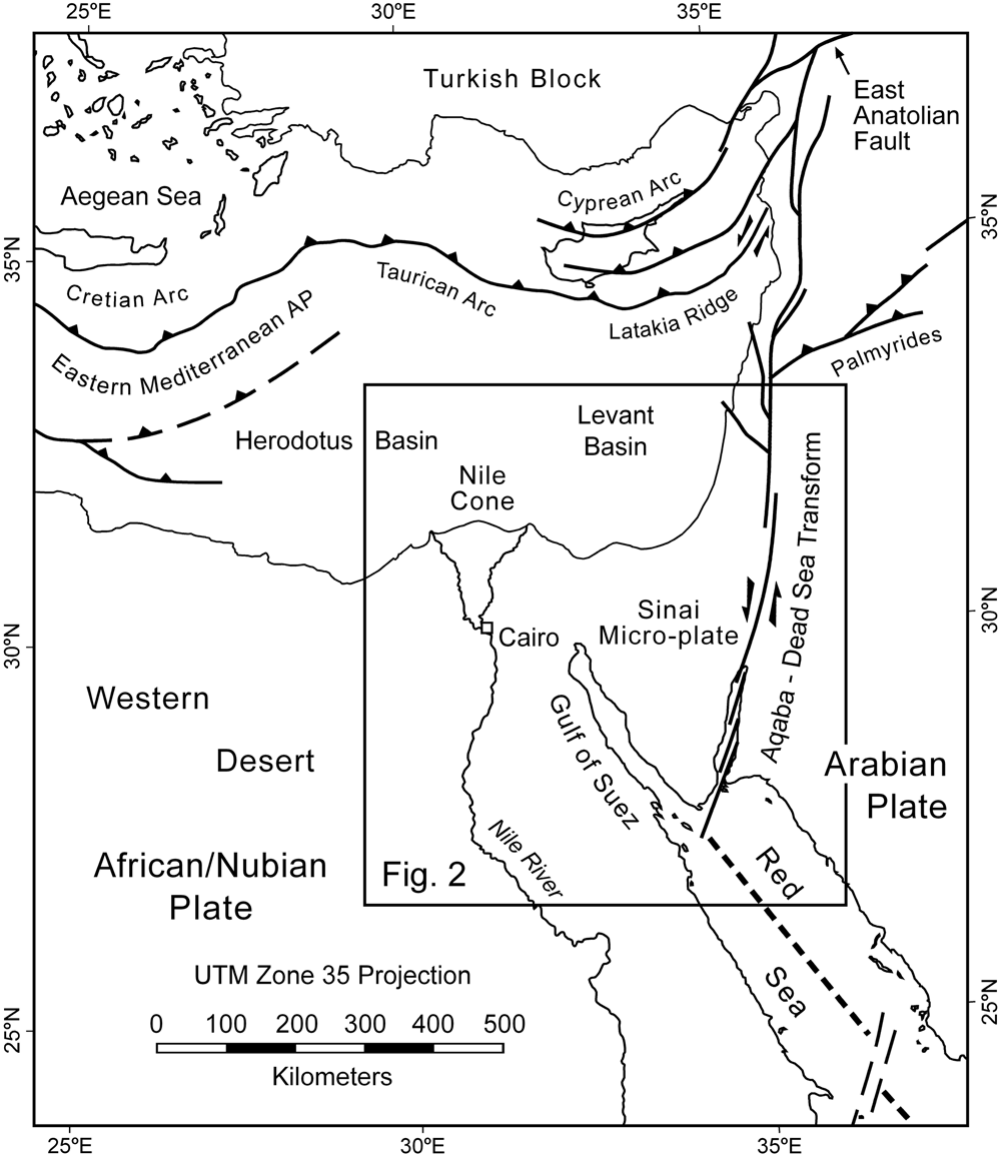
Tectonic setting of the Gulf of Aqaba – Dead Sea sinistral transform fault. Plate boundaries are shown with bold lines^65–67^. AP = accretionary prism. Figure was generated with Macromedia FreeHand MX v11.0.1 (https://www.adobe.com/mena_en/products/freehand/). Location of Fig. 2 is indicated.

**Figure 2:**
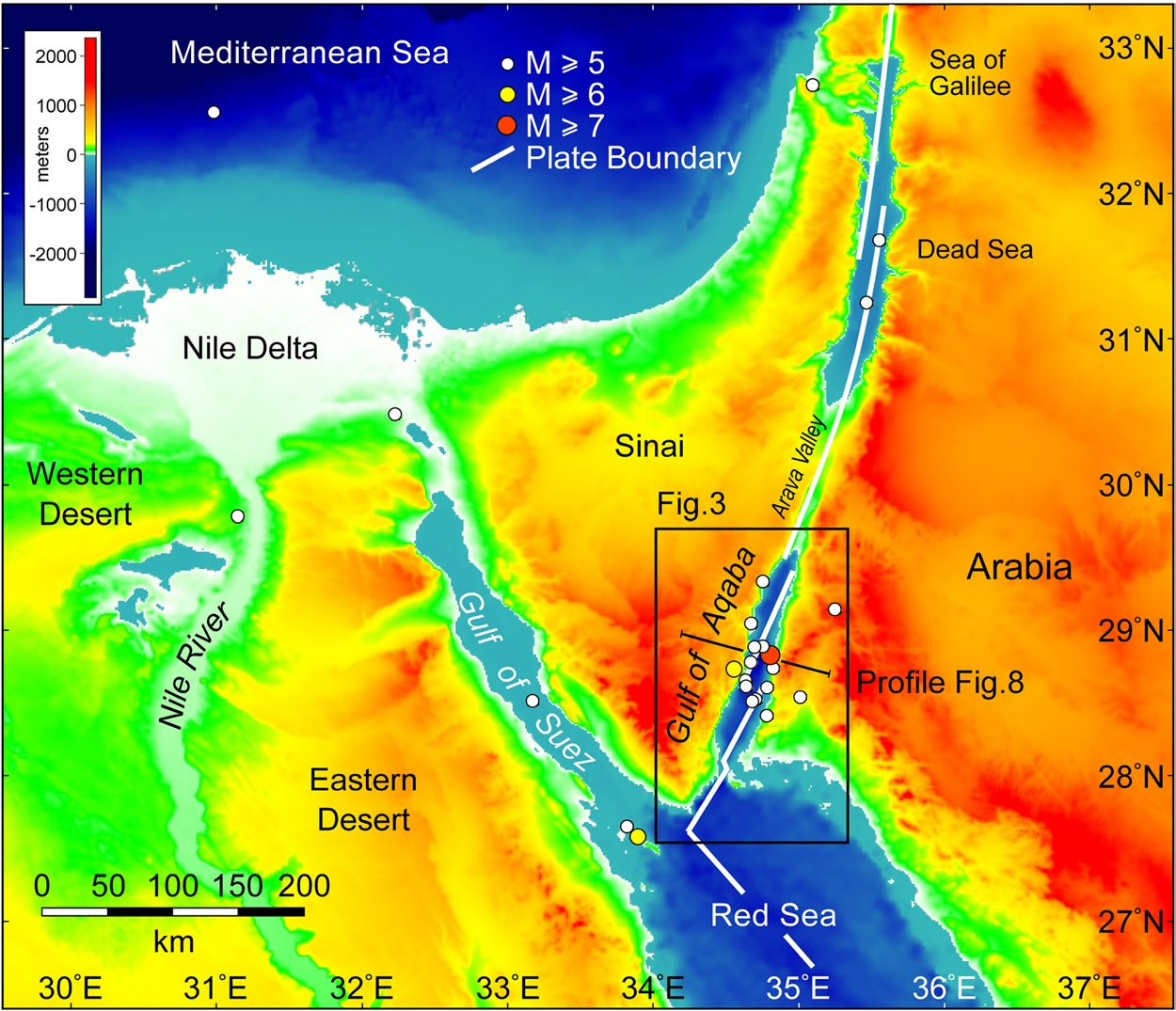
Digital elevation and bathymetric model for the Gulf of Aqaba and environs^47^. Epicenters for earthquakes with M ≥ 5 recorded from 1 January 1960 to 1 June 2016 are shown with colored dots^14^. Basemap was generated with Global Mapper v13.00 (http://www.bluemarblegeo.com/products/global-mapper-download.php). Location of Figs 3 and 8 are shown.

The Gulf of Aqaba and Dead Sea fault system originated in the Miocene as a transform plate boundary linking the northern Red Sea to the East Anatolian fault and Zagros-Bitlas convergence zone in eastern Turkey^1–5^ (Figs 1 and 2). This effectively ended continental rifting in the Gulf of Suez. A variety of different datasets indicates that transform initiation occurred at ~14–11 Ma^6, 7^. With 107 km of total sinistral offset^8^ this gives average slip rates of 0.76–0.97 cm/yr. The geodetically estimated present-day slip rate for the southern Dead Sea Rift^9^ is only about half of this at 0.44 ± 0.03 cm/yr. Other estimates have suggested 0.5–0.7 cm/yr for the past 5 Myr^10, 11^. Even considering observational uncertainties these interpretations allow for the possibility that slip rates on the Gulf of Aqaba-Dead Sea transform have varied significantly through time.

**Figure 3:**
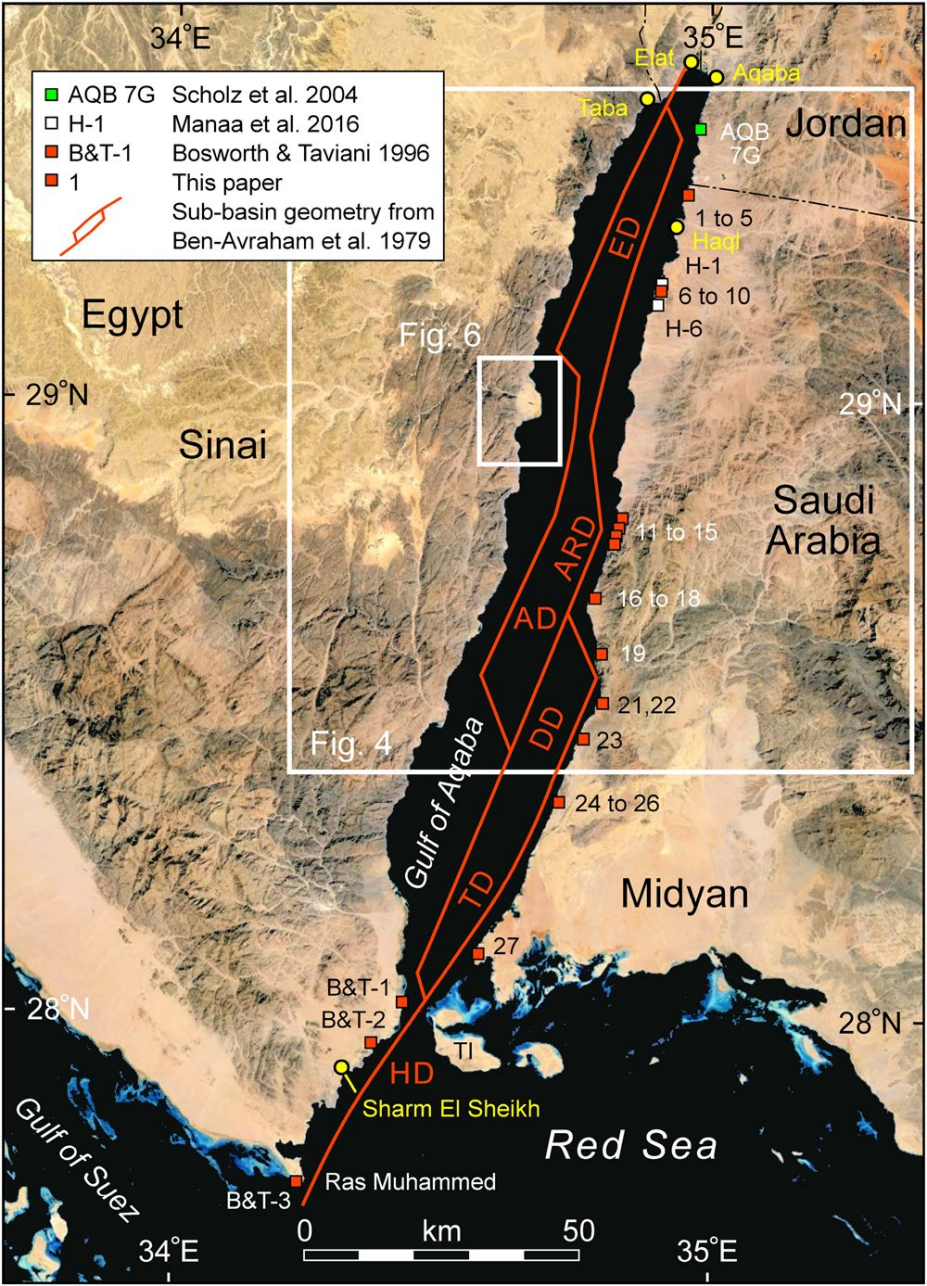
Last interglacial (Marine Isotope Substage 5e) coral terrace and fault kinematic field stations in the Gulf of Aqaba and southern tip of Sinai. Internal sub-basins and main fault geometry are shown with red lines^1^. Location of Figs 4 and 6 are indicated. HD = Hume Deep; TD = Tiran Deep; DD = Dakar Deep; AD = Arnona Deep; ARD = Aragonese Deep; ED = Elat Deep; TI = Tiran Island. Basemap data: Google, SIO, NOAA, US Navy, NGA, GEBCO; Image Landsat.

Much of the Gulf of Aqaba region is relatively unpopulated and with little infrastructure development. However large cities exist at the north end of the Gulf: Taba (Egypt), Elat (Israel), and Aqaba (Jordan) (Fig. 3). Sharm el Sheikh at the confluence with the Red Sea has grown rapidly in recent decades, and several smaller communities now exist along both margins of the Gulf. A large teleseismic earthquake in November of 1995 located offshore in the central sub-basin resulted in one fatality, numerous injuries, and the local destruction of buildings over a broad area^12^. As human development of the Gulf of Aqaba plate boundary and its environs progresses it is becoming increasingly important to better quantify future seismic risks for the region. In this paper we integrate seismological, outcrop, and geochronological data to assess the distribution of tectonic deformation through time and space in the Gulf of Aqaba. Our primary dataset is elevations of uplifted coral terraces that formed during the last interglacial sea-level high-stand. We also consider the longer-term uplift of the Arabian basement complex. This suggests a model in which seismicity will remain focused in the central Gulf of Aqaba sub-basin. Slip in the northern and southern sections of the basin will be accommodated aseismically or by smaller, non-teleseismic earthquakes

.

### Gulf of Aqaba Seismicity

**Figure 4:**
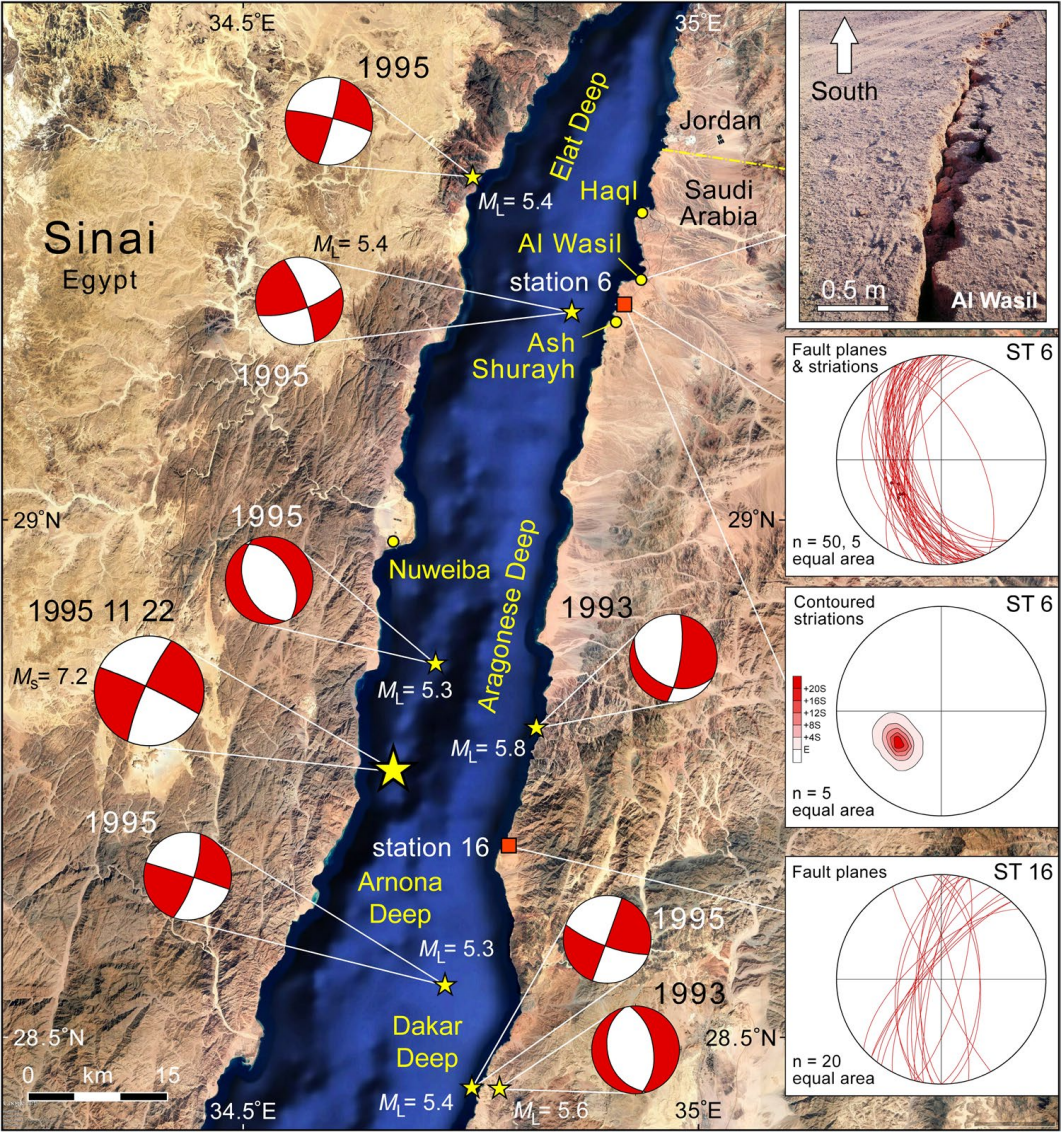
Earthquake focal mechanisms and magnitudes of the November 22, 1995 7.2 *M*_*S*_ sinistral strike-slip earthquake (larger symbol), its largest aftershocks, and the two largest events in the 1993 seismic swarm^15^. Several of the events displayed predominantly normal fault mechanisms. The outcrop photograph from near Al Wasil is of a fissure with ~30 cm dip-slip extensional offset (photographed December 2005). Stereoplots are outcrop measurements of small-scale extensional faults from coral terrace station areas 6 and 16 (Fig. 3). In area 6 the faults cut partially lithified Plio-Pleistocene sandstone and conglomerate beds. The average slip direction of the limited kinematic data plunges 46° toward 231° (ENE-WSW extension). No strike-slip faults were observed. The measurements for area 16 are from within the Pleistocene coral terrace but below the actual MIS5e high-stand. No kinematic data were observed at this station but if dip-slip is assumed then the extension was approximately east-west. Location of map is shown in Fig. 3. Basemap data: Google, SIO, NOAA, US Navy, NGA, GEBCO; Image Landsat.

Compilations of seismicity along the Gulf of Aqaba-Dead Sea transform indicate a very low seismic efficiency and therefore the high importance of aseismic creep^13^. Larger earthquakes, in particular those with *M*_L_ ≥ 5, are concentrated in a very tight cluster around the Aragonese-Arnona deep^1^ and then in an elongate belt that starts at about 31°N and continues north through Israel, Jordan and Lebanon (Fig. 2)^14^. At least six *M*_L_ 5.2 to 6.1 events occurred within the Aragonese-Arnona deep between February 1983 and May 2016^12–15^. The largest took place on 22 November 1995 and had an interpreted *M*_S_ of 7.2^14, 15^. This earthquake had a strike-slip focal mechanism and was located offshore and south of Nuweiba, Egypt (Fig. 4). Buildings were damaged in Haql and Ad Durrah (Saudi Arabia), Nuweiba, Aqaba, and Elat^12, 16^. The relatively small number of injuries that occurred was largely attributed to the absence of high-rise buildings.

Most of the teleseismic events recorded for the Gulf of Aqaba-Dead Sea transform boundary have yielded strike-slip fault solutions whenever sufficient data have been available. However normal fault mechanisms are observed in local network recordings^12, 15^. Composite focal mechanisms derived from events that occurred from 1985–1989 with *M*_L_ 2.6 to 3.8 indicated normal dip-slip on approximately north-south striking faults in the northern Aragonese and eastern Elat Deeps^17^. Similarly aftershock studies of the large 1995 Nuweiba event show that the total deformation field was complex and involved more than just sinistral strike-slip on the western boundary fault of the Aragonese-Arnona deep^12, 15^ (Fig. 4). Although strike-slip mechanisms predominated in the larger aftershocks, one large normal fault event occurred in the offshore north of the main epicenter. In 1993 an earthquake swarm of over 420 events occurred within and nearby the Aragonese and Dakar deeps^15^. The two largest events of the swarm (*M*_L_ = 5.8 and 5.6) were principally dip-slip (Fig. 4). The epicenters of both of these earthquakes are located on NNE-SSW striking segments of the Arabia coastline adjacent to the Dakar and Aragonese Deeps. This is the general orientation of the Gulf of Aqaba–Dead Sea transform boundary and such structures would usually be associated with sinistral strike-slip movement. In this case the seismic events document a component of east-west extension. The recognition of a small component of Gulf normal opening led to the early designation of this plate boundary as a “leaky transform”^1^.

There has been considerable discussion about why the levels of seismicity are presently so low north of the Gulf of Aqaba in the Arava Valley^18^ (Fig. 2). Analysis of surface ruptures observed in trenches shows that the transform boundary has switched between periods of relatively high and relatively low seismic activity for at least the past 5 kyr^19^. The two modes display recurrence intervals of ~200 yr and ~500 yr, respectively. Further north in the area of the Dead Sea (Fig. 2), observations of breccia beds in lacustrine strata indicate that with respect to *M* ≥ 7 earthquakes the seismic regime has been essentially constant for at least the past 40 kyr^20^. Prior to this at ~50 ka there was a strong clustering of *M* ≥ 7 earthquakes with the recurrence interval then increasing logarithmically to its present value of ~11 kyr. Similar datasets are not available for the Gulf of Aqaba.

The southern Gulf of Aqaba has not experienced any instrumentally recorded *M* ≥ 5 earthquakes^14^ (Fig. 2). In regard to recent seismicity this segment of the transform boundary therefore resembles the north. The pattern changes in the Gulf of Suez, which was once the northernmost segment of the Red Sea. The southern Gulf of Suez is presently only extending at ~0.1 cm/yr, but nonetheless the area is seismically active^21–23^ (Fig. 2). Teleseismic events were recorded on 31 March 1969 (*M*_w_ = 6.6) and 28 June 1972 (*M*_w_ = 5.6)^14, 21^. Unlike the Nuweiba 1995 earthquake these were predominantly normal fault events. For the region of Fig. 2 the present frequency of *M* ≥ 5 earthquakes in the southern Gulf of Suez is only exceeded by that occurring in the Aragonese-Arnona/Dakar Deeps themselves.

## Methods

### Outcrop Fault Kinematics

Numerous surface breaks have been reported along the margins of the central Gulf of Aqaba in association with the region’s seismicity^12^. The best documented structures were associated with the 22 November 1995 earthquake and its aftershocks. Near Ash Shurayh on the Saudi margin several large fissures were recorded, striking approximately N-S and cutting unconsolidated gravels of the narrow coastal plain (Fig. 4). Each fissure was several hundred meters in length with 0.25–0.3 m dip-slip movement down toward the Gulf of Aqaba. Aligned meter-scale sand volcanos were also observed. Similar extensional fissures associated with a pre-December 2005 earthquake are also present along the coastal plain near Al Wasil (Fig. 4).

**Figure 5:**
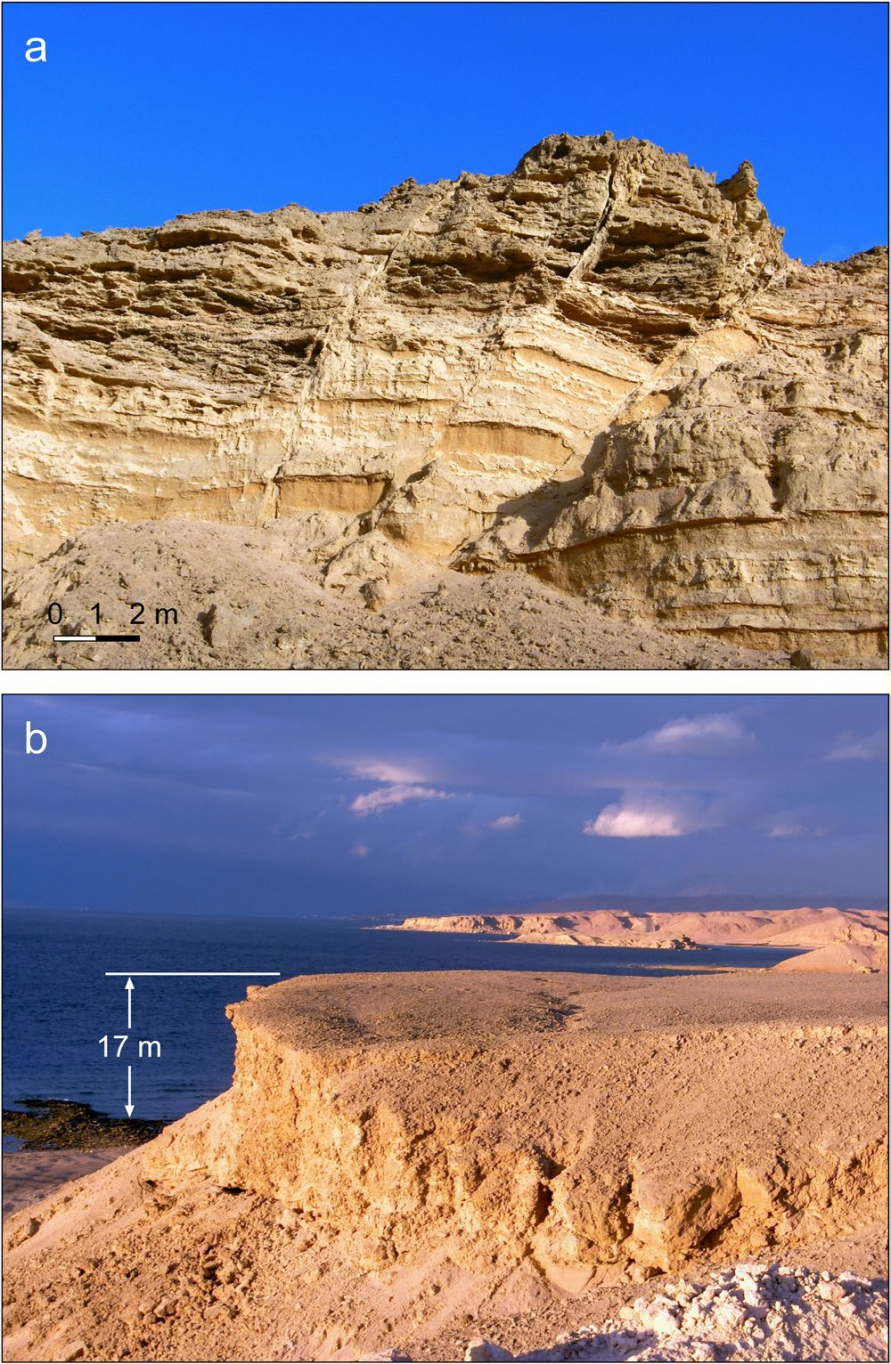
Examples of the Plio-Pleistocene exposures described in this study near Haql, Saudi Arabia (station 6 in Figs 3 and 4). (**a**) Extensional faults cutting partially lithified sandstone and conglomerate stratigraphically beneath the Pleistocene terraces of the coastal region. (**b**) Last interglacial (Marine Isotope Substage 5e) coral terrace. Terrace is approximately 17 m above sea level at this location.

These fissures affected recent, unconsolidated sediments and could simply indicate gravity-driven mass movement of coastal material toward the offshore topographic basin triggered by the earthquakes. In this case they would not directly reflect the regional upper crustal-scale stress field. However numerous basement-involved extensional faults are present along both margins of the Gulf of Aqaba, generally striking N-S to NNE-SSW. Few of these faults display strike-slip kinematic indicators. Rather, most show evidence of mostly dip-slip movement. The age of faulting is generally unconstrained except that it is post-Neoproterozoic. Similar striking faults are also present in the sandstone and conglomerate sequence that underlies the coastal plain alluvium and is well-exposed in the vicinity of Al Wasil (Fig. 5a). The strata are undated, except that they are post-Miocene and pre-date the Pleistocene coral terraces that we describe below. The fault movement is therefore relatively young. The detailed geometry of these faults suggests simple dip-slip movement, which is supported by the several instances where good slickenside striations are present (Fig. 4). The slip direction suggests ENE-WSW extension in a normal fault stress regime.

Younger small-scale faults are also observed within different levels of the Pleistocene coral terraces themselves. Abundant faulting at our Station 16 (Fig. 4) does not preserve any true kinematic markers but if pure-dip slip is assumed then they would also indicate E-W extension.

**Figure 6:**
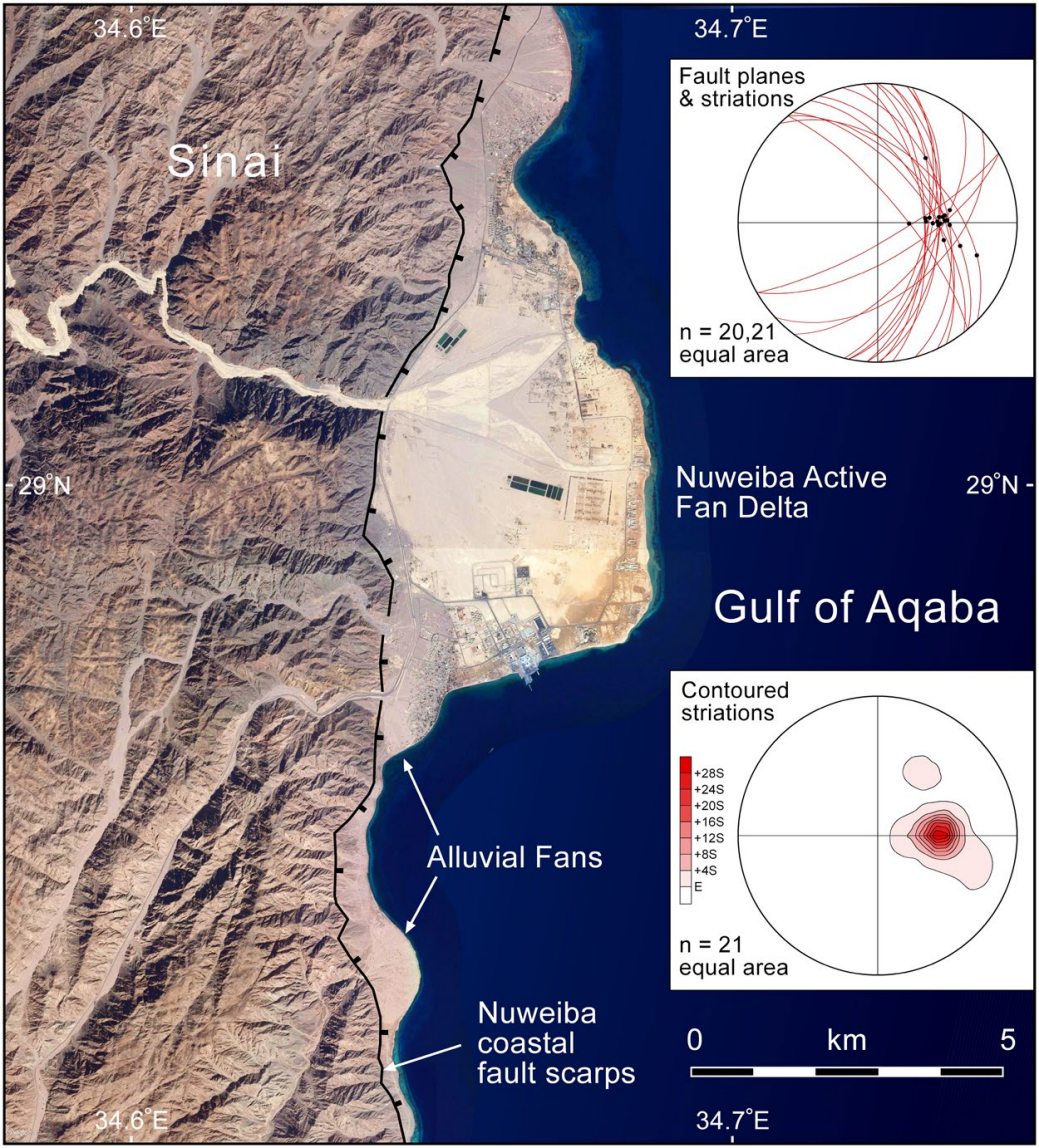
Outcrop measurements of the Nuweiba coastal fault system on the Sinai coastline. 20 stations were recorded; at one location two different sets of striations were observed. All faults cut partially lithified conglomerate and sandstone deposited in active alluvial fans. The average slip direction plunges 56° toward 090° (east-west extension). No strike-slip faults were observed; a few had minor oblique-slip component. Location of map is shown in Fig. 3. Basemap data: Google, SIO, NOAA, US Navy, NGA, GEBCO; Image Landsat.

Even more remarkable examples of young extensional faulting are present along the Sinai coastline in the area of Nuweiba (Fig. 6). The coastline is marked by an active fault scarp with Neoproterozoic basement rocks in the footwall and active Holocene semi-consolidated alluvial fan sandstone and conglomerate in the hanging wall^24^. The overall fault system strikes N-S with several oblique segments linking the main fault strands. Slickenside striations and larger mullions commonly decorate the fault surfaces and provide excellent kinematic control (Fig. 6). Dip-slip movement overwhelmingly predominates and indicates E-W extension, similar to the young extensional faults on the Arabian margin. No “neotectonic” strike-slip faults have been documented in outcrop on the Sinai margin of the Gulf of Aqaba.

Field structural measurements demonstrate that at least in post-Miocene times E-W to ENE-WSW extension has dominated along the exposed margins of the central Gulf of Aqaba. Strike-slip faulting may also occur but such faults with constraints on the timing of movement have not yet been reported. The extensional faulting predominantly strikes parallel to the overall regional trend of the transform boundary^1, 24, 25^, rather than highly obliquely as would be the case in a traditional pull-apart basin model^26, 27^.

### Pleistocene Coral Terrace Elevations

Much of the coastline of the Red Sea, Gulf of Aqaba and Gulf of Suez is fringed by coral reefs^28^ (Fig. 5b). Similar constructional biohermal features were present in these same areas throughout most of the Pleistocene^29^. The interplay of eustasy and tectonism has resulted in elevations from at least 60 m below to more than 320 m above present sea level for these ancient terraces^30, 31^. All of these reef terraces are potential paleo-sea-level markers, if their age can be determined. The best preserved and least diagenetically altered terraces are those that were formed during the last interglacial in Marine Isotope Substage 5e (MIS5e; ~125 ka)^32^. For these reasons, and because eustatic sea level was higher than today, the MIS5e terraces have been the subject of most tectonostratigraphic interpretations^23, 32–39^.

Studies of MIS5e coral terraces in tectonically stable domains have generally found the last interglacial sea level to have been ~5–10 m higher than present-day^40–42^. These are local sea-level estimates, which must be synthesized to determine a value for global sea level. This is a complex process, but fortunately the last interglacial dataset is fairly robust. A detailed probabilistic and physical modeling approach has suggested that the MIS5e global sea level was 7.2 ± 1.3 m (67% confidence level) higher than present-day^43^.

U-series dated MIS5e coral terraces in the southern Gulf of Suez lie as much as 18.5 m above sea level with net tectonic uplift of ~11.5 m (~0.01 cm/yr)^23^. This uplift has been shown to be the flexural response to movement on individual extensional fault segments with active lengths of a few tens of kilometers^22, 23^. MIS5e terraces in the Gulf of Aqaba were similarly reported to occur at 19 m elevation or more^44^, although the details of the structural setting were not discussed. Very high MIS5e terraces may occur on Tiran Island at the southern end of the Gulf of Aqaba (Fig. 2). A coral from an elevation of 40 m was Uranium-series dated as ~130 ka although the analytical systematics and location of this sample were not reported^45^.

In order to obtain as much information as possible from a field program along the Saudi Arabian Gulf of Aqaba coastline we elected to integrate Light Detection and Ranging (LIDAR) topographic surveying of coral terrace exposures with detailed stratigraphic measurements, petrographic and mineralogical screening and ^230^Th/U dating. We mapped representative stations of all the coral terraces exposed from the Saudi border with Jordan to the Midyan region at the southern end of the Gulf (Fig. 3). We present here only data for the terraces that would subsequently be interpreted to have formed during the last interglacial at ~125 ka. This resulted in 27 site elevations (Table 1). The LIDAR sites were chosen so that all measurements could be made relative to the local sea level at the time the site was occupied. The elevation of one terrace was measured by laser distance range finder as the terrace was immediately overhanging the shoreline. For the LIDAR stations, 10 measurements from the 3D elevation cloud were averaged for each site (Table 1). The standard deviation for these averages reflects the rugosity of the terrace surface itself and not the accuracy of the LIDAR measurement. No corrections for tidal variations were made as the Gulf of Aqaba is a micro-tidal environment and tide charts are not readily available for all locations along the coast. The tidal range at the north end of the Gulf at Elat and Taba is usually about 90 cm. Similar assumptions were made by previous workers in this basin and other parts of the Red Sea system. This does add an additional uncertainty regarding each specific site elevation but the robustness of our dataset helps ameliorate this and we focus here on overall trends within the dataset.

**Table 1:** Last interglacial (MIS5e; ~125 ka) coral terrace stations along the eastern margin of the Gulf of Aqaba and southeastern Sinai.

Station Name	Average or Spot Elevation (m)	Standard Deviation/Uncert	Longitude	Latitude
AQB 7G	10	NA	34.97047055° E	29.44777656° N
1	10.74	0.94	34.95715423° E	29.35555275° N
2	13.42	0.72	34.95761584° E	29.35545575° N
3	11.51	0.63	34.95634038° E	29.35069241° N
4	15.81	0.40	34.95509023° E	29.34994381° N
5	14.5	0.5	34.94922600° E	29.33866200° N
6	17.43	0.22	34.90029423° E	29.19107390° N
H-1	19.2	NA	34.90061667° E	29.19083333° N
7	17.29	0.55	34.90028840° E	29.19075817° N
8	16.92	0.42	34.90028840° E	29.19075817° N
9	19.12	0.61	34.90036625° E	29.18940360° N
10	19.88	0.49	34.90041765° E	29.18940287° N
H-6	16.1	NA	34.89296667° E	29.16123333° N
11	19.80	0.48	34.82639797° E	28.79335298° N
12	19.38	0.27	34.81950761° E	28.78000057° N
13	23.45	0.42	34.81953531° E	28.77864663° N
14	25.21	0.56	34.81663724° E	28.76803754° N
15	22.61	0.42	34.79744916° E	28.71207527° N
16	24.51	0.63	34.78177513° E	28.67808170° N
17	22.75	0.50	34.78180414° E	28.67677283° N
18	19.24	1.80	34.77984360° E	28.67580598° N
19	24.88	0.64	34.79299324° E	28.58561747° N
20	17.89	0.79	34.79558864° E	28.50662182° N
21	20.37	0.25	34.79567779° E	28.50585358° N
22	22.43	0.43	34.79411443° E	28.50402427° N
23	26.09	0.45	34.76021296° E	28.44950954° N
24	17.49	0.30	34.71886827° E	28.34571462° N
25	17.42	0.34	34.71890255° E	28.34467636° N
26	17.87	0.14	34.71833364° E	28.34418718° N
27	8.73	0.24	34.57208808° E	28.09622663° N
B&T-1	9.1	0.5	34.43766667° E	28.01750000° N
B&T-2	11.2	0.5	34.38233333° E	27.95016667° N
B&T-3	3.5	0.5	34.24883333° E	27.72450000° N

Sites are listed from north to south. NA indicates that no measure of elevation uncertainty was provided in the source publication. AQB 7G is the only location in Jordan and is from ref. 46. H-1 and H-6 are from ref. 44. B&T-1, 2 and 3 are located on the southeast coast of Sinai, Egypt^23^. Our elevations are local relative measurements directly from sea level and uncorrected for tides. Datum is WGS84. Locations are shown in Fig. 3.

In addition to the 27 new terrace elevation sites we have also incorporated measurements that two of the authors previously made along the southeast margin of Sinai^23^. These elevations were determined with surveyor’s tape and clinometer with reproducibility of ±0.5 m. As with our LIDAR data, these measurements are elevations above local sea level uncorrected for tides. Published location and elevation information were also available for three sites near the Saudi-Jordan border^44, 46^. Uncertainties in these elevation measurements were not reported.

### Uranium-series Dating

Material for Uranium-series dating was collected from each of the 27 raised Pleistocene coral terrace sites and analyzed at the Laboratoire des Sciences du Climat et de l’Environnement (Gif-sur-Yvette, France) (See Supplementary Material). Depending on the terrace stratigraphy, coral and botryoidal aragonite samples were collected at different levels (from the base to the top), corresponding to different reef facies (i.e. reef slope, reef crest, reef flat, etc.).

Seven samples from the Saudi Gulf of Aqaba margin produced Uranium-series dates that are clearly associated with the MIS5e last interglacial sea-level high-stand (Table 2). These are in agreement with the results of previous studies along this margin^32, 44, 46^. Although many sites did not yet provide reliable MIS5e dates, lateral continuity of the reef terraces demonstrates that they belong to the same geomorphological structure (Fig. 5b). We can therefore confidently include all 27 sites within our analysis of uplift during the past 125 kyr. Terraces at elevations higher than those of the MIS5e were also measured and dated. These are much less continuous laterally than the MIS5e and display much more severe diagenetic alteration. These attributes make them readily discernible from the MIS5e terraces in the field.

**Table 2:** Uranium-series and XRD measurements for samples collected within the strata of MIS5e terraces along the Saudi Arabian coast of the Gulf of Aqaba.

Station Name	^238^U (µg/g)		^232^Th (ng/g)		δ^234^U_m_		(^230^Th/^232^Th)		(^230^Th/^238^U)		Age (ka)		δ^234^U_(0)_		Age (ka)*		Arag (%)	Calc (%)
9	2.228	±0.001	0.097	±0.001	107.7	±0.9	56513	±160	0.763	±0.002	123.93	±0.83	152.81	±1.26	121.54	±0.84	99.2	0.8
14	2.072	±0.001	0.191	±0.002	118.8	±1.6	28058	±105	0.815	±0.003	137.19	±1.42	175.10	±2.41	125.56	±1.48	99	1
15	5.013	±0.007	0.366	±0.002	108.9	±1.7	32881	±123	0.755	±0.003	121.19	±1.19	153.36	±2.45	118.58	±1.45	100	
20	2.656	±0.003	0.060	±0.001	106.3	±2.1	108125	±610	0.764	±0.004	124.48	±1.77	151.13	±3.10	122.76	±1.96	100	
22	2.774	±0.002	0.074	±0.001	114.5	±1.3	91669	±358	0.768	±0.003	123.69	±1.18	162.39	±1.98	117.42	±1.23	99.3	0.7
27	2.307	±0.001	0.196	±0.001	107.2	±0.9	28838	±107	0.763	±0.003	123.86	±1.04	152.17	±1.32	121.73	±0.96	97.6	2.4
27	2.585	±0.002	0.145	±0.001	107.8	±1.0	43212	±190	0.758	±0.003	122.36	±1.20	152.39	±1.53	120.14	±1.11	98.4	1.6

Location coordinates are given in Table 1. ^238^U and ^232^Th concentrations were determined using the enriched ^236^U and ^229^Th isotopes, respectively. δ^234^U_m_ = {[(^234^U/^238^U)_sample_/(^234^U/^238^U)_eq_] − 1} × 1000, where (^234^U/^238^U)_sample_ is the measured atomic ratio and (^234^U/^238^U)_eq_ is the atomic ratio at secular equilibrium. δ^234^U_(0) _is the initial value and is calculated by the equation: δ^234^U_(0)_ = δ^234^U_meas_ exp^(λ234t)^, where t is the age in years and λ_234_ is the decay constant for ^234^U (ref. 69). *Corrected ages calculated using the open-system model^70^ and present-day seawater δ^234^U value of 146.8‰ (ref. 71). Dr. W. Thompson kindly provided the Excel spreadsheet to calculate open-system ages. Arag (%) and Calc (%) are percentage of aragonite and calcite obtained by X-ray diffraction. See Supplementary Information for analytical procedures. Stations 9, 14, 20, 22, 27: coral samples; Station 15: botryoidal aragonite.

## Results and Discussion

**Figure 7:**
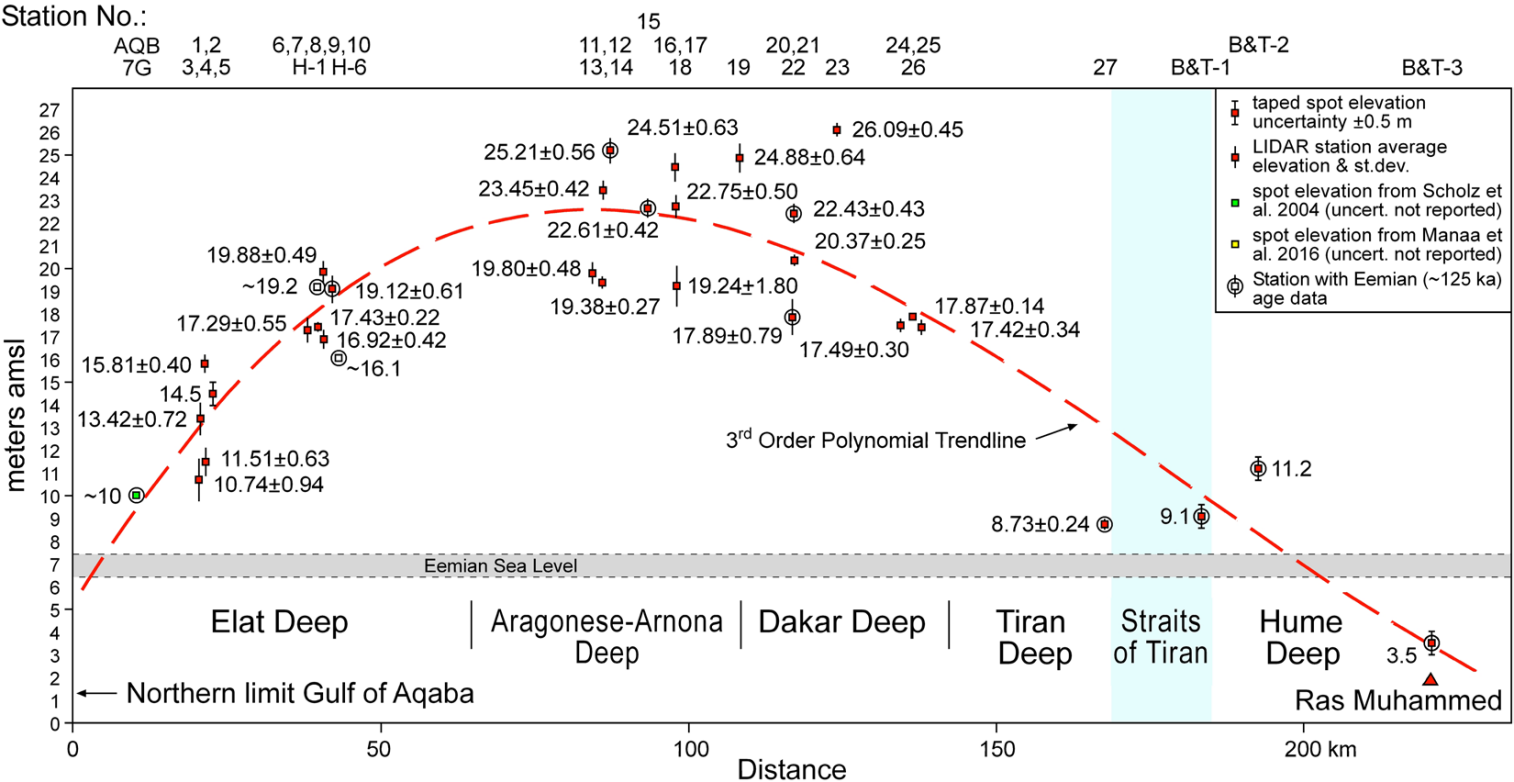
Elevation profile connecting the station locations of Fig. 3 (see also Table 1). All elevations are relative to local sea level without a tidal correction. Dashed curved line is a 3^rd^ order best-fit polynomial trendline. Elevations are for the top of the terrace. Vertical bars give uncertainty of elevation measurements (standard deviation of 10 measurements at LIDAR stations; 0.5 m for taped or laser range finder stations; no error bar for sources where none was given). Stations with Uranium-series dates are shown with outer circles and are described in Table 2 or taken from the literature.

The locations of the field survey sites that produced the elevation data of Table 1 are shown in Fig. 3. These data are plotted in Fig. 7 as a structural profile that runs along the eastern margin of the Gulf of Aqaba. Most of the sites show net tectonic uplift above the MIS5e sea level (+7 m above present-day; shaded band in Fig. 7). Variation in terrace elevations at very closely located sites may have several causes. For example Stations 20 to 22 show a range of about 17.9 to 22.4 m. This may be due to local tilting of the terrace surface or the presence of very small offset extensional faults similar to those described above. It might also reflect different degrees of erosion of the top of the original coral terrace. We are investigating these details but here our emphasis is on the regional trend in MIS5e terrace elevation.

Our observations agree with previous reports that MIS5e terraces at the very north end of the Gulf of Aqaba lie at elevations of ~10 m (Fig. 7)^46^. Terrace elevation then increases toward the south to the area adjacent to the Aragonese and Dakar Deeps where the highest values are ~25–26 m. Further south the elevations systematically decrease until at Ras Muhammed on the Sinai coast they are ~3.5 m above sea level. Unlike the coastlines of the Gulf of Aqaba, Ras Muhammed has experienced net tectonic subsidence during the past 125 kyr^23^. A 3^rd^ order polynomial fit to our terrace elevation dataset illustrates the general vertical response of the eastern Gulf of Aqaba margin to deformation since the last interglacial (Fig. 7).

**Figure 8:**
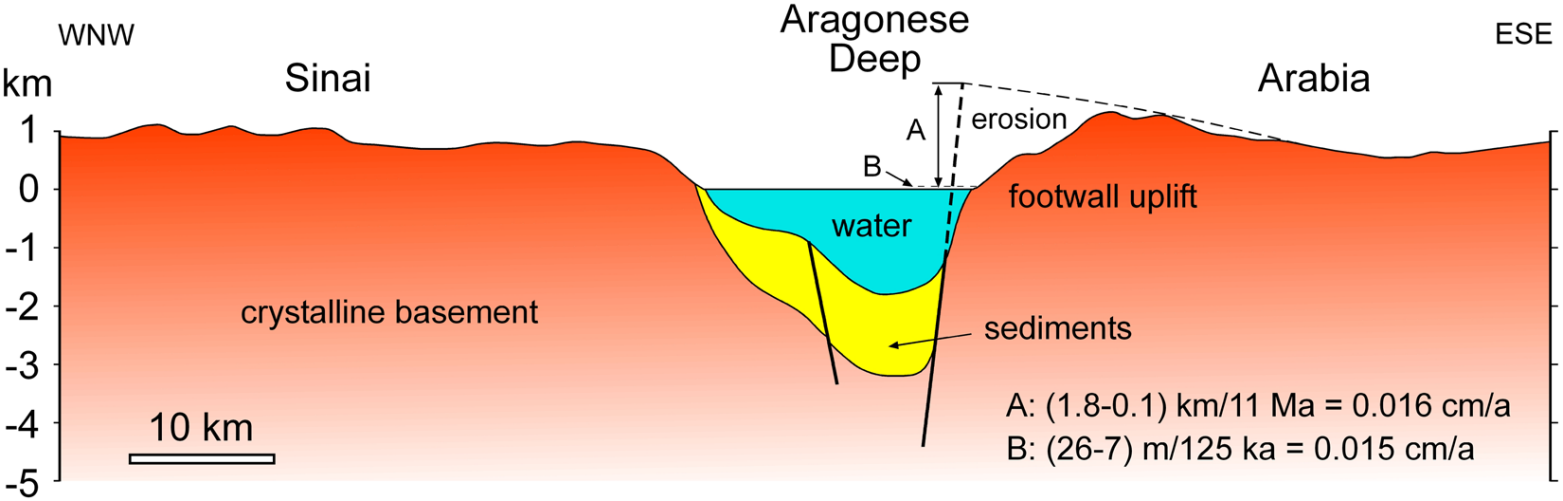
Topographic and bathymetric profile across the deepest part of the Gulf of Aqaba (Aragonese Deep) in the general vicinity of the 22 November 1995 *M*_S_ 7.2 earthquake^14, 15^ and our coral terrace station 12 (Figs 2 and 3 and Table 1). Structure of the Aragonese Deep is modified from ref. 48. The east Aragonese Deep bounding fault complex has a large extensional component which drives the footwall uplift. Two uplift rate calculations are shown: A–uplift since the minimum age of initiation of the Gulf of Aqaba at 11 Ma (assuming paleo-sea-level 100 m higher than today); and B–uplift since the MIS5e interglacial at 125 ka (assuming paleo-sea-level was 7 m higher than today).

Figure 8 displays a topographic-bathymetric profile across the Aragonese Deep based on published digital elevation models^47^ and a slightly modified interpretation of the subsurface geology^48^. Major strike-slip faults occur along both sides of this deep, although the eastern fault also displays a very large dip-slip component^1, 5, 48^. Taking a maximum terrace elevation of ~26 m, minus the higher MIS5e sea-level of 7 m, this yields a tectonic uplift of 19 m and an average uplift rate of 0.015 cm/yr for the past 125 kyr.

The Gulf of Aqaba – Dead Sea plate boundary’s east-west extension and leaky transform character is well-documented^1–3, 24, 25^. The uplift of the margins of the Gulf of Aqaba is not due to the dominant deformation mode of the transform boundary which is clearly sinistral strike-slip faulting. It is also not caused solely by the long-recognized left-stepping nature of the internal Gulf sub-basins^1–5^. The oblique-striking faults that help define these steps are not as active as the basin margin normal faults and 2D reflection seismic profiling suggests that at least the link between the eastern and western Elat deep bounding faults (Fig. 3) is via a smooth, arcuate fault rather than a discrete step^49^. Uplift is strongest on the Arabian margin in the vicinity of the step between the Dakar Deeps and Arnona-Aragonese Deeps (Figs 2, 3 and 7). However basement uplift is present along most of the Sinai margin and significant uplift has also occurred far from this step along the eastern (Arabian) side of the Elat Deep. We suggest that most of the observed uplift is generated by the margin-parallel (N-S to NNE-SSW) young extensional faults that are present in outcrop and commonly experience low magnitude dip-slip earthquake events (Figs 4 and 6).

The extensional faulting critical to our uplift model is focused onshore along both shoulders of the rift, while present-day strike-slip faulting and associated large magnitude earthquakes are almost exclusively offshore along or close to the rift axis. This observed “strain-partitioning” may not have been as prevalent in earlier phases of the evolution of the Gulf of Aqaba as undated N-S strike-slip faults are present in the basement complex of the Arabian margin^50^. These strike-slip faults indicate that at some time in the past strike-slip faulting was distributed across a broader region than is occurring in the present deformation field.

We do not have reliable sea-level estimates for older features along the margin, but a first-order estimate can be made for the Arabian crystalline basement. The land surface at the northern end of the Red Sea was near to paleo-sea-level at the beginning of rifting ~23 Ma, as shallow marine deposits are recorded shortly after rift initiation^6^. Most of the pre-rift stratigraphic section had been previously eroded away in the area where the Gulf of Aqaba would develop, with minor occurrences preserved in local fault-bounded inliers^51^. Although Red Sea rift shoulder uplift began soon after rift initiation^52, 53^, the region of the future Aragonese Deep was tens of kilometers in-board from the active rift margin. We assume that the basement in the central Gulf of Aqaba remained nearly at sea level into the Middle Miocene, with minor uplift and erosion keeping pace. In Fig. 8 the high corner of the Aragonese Deep footwall block that was removed by erosion during post-Aqaba rift-initiation uplift has been restored. This is a simple projection of the rift shoulder that reflects the flexural rigidity of the crust and is a minimum estimate of the material that has been removed. The interpreted 1.8 km of uplift occurred since onset of strike-slip and associated secondary extensional movement on the Gulf of Aqaba transform boundary at 14–11 Ma. Paleo-sea-level at 14–11 Ma was thought to be no more than ~50–100 m above present-day^54^ though more recent analyses suggest it may have only been ~25 m above present^55^. We therefore decrease our net tectonic uplift by 100 m to 1.7 km to cover the more extreme scenario. Using the range of initiation ages for the Gulf of Aqaba faulting (14–11 Ma) uplift rates of 0.012–0.016 cm/yr are obtained.

The basement uplift model of Fig. 8 is very conceptual and the assumptions are somewhat simplistic. It is very interesting however that this simple analysis yields uplift rates, and by inference, similar E-W extensional rates for the region of the Aragonese Deep for both the past 125 kyr and the preceding 14–11 Myr. Comparison of Figs 2 and 7 shows that footwall uplift and the strong seismicity of the Arnona-Aragonese Deep are coincident. Strike-slip faults are accomplishing the dominant relative plate motion, but they are spatially associated with extensional faults that drive local uplift. Because present-day seismicity is focused where uplift is greatest, we suggest that high levels of seismic activity have been focused in this region for at least the past 125 kyr, and perhaps since strike-slip initiation at 14–11 Ma.

Several mechanisms have been proposed to explain mechanically why the footwall blocks of extensional faults are uplifted coevally with the subsidence of their corresponding hanging wall blocks. Models invoking isostatic rebound^56–58^, cantilever effects^57^, lithospheric mantle delamination^59^, and bending of a multilayer crust with variable visco-elastic-plastic properties^60, 61^ have been applied to many continental rift settings. But why should extension be occurring across the Gulf of Aqaba–Dead Sea transform in the first place? Ben-Avraham and Zoback^25^ assessed the orientation of the principal horizontal stresses S_Hmax_ and S_hmin_ adjacent to a strike-slip fault for various values of the ratio of the frictional strength of the fault versus the shear stress at failure in the adjacent crust. If the fault is considerably weaker than the adjacent crust, then one or the other of the principal horizontal stresses will rotate toward parallelism with the fault as distance from the fault is decreased. In a divergent plate setting (far-field S_Hmax_ < 45° to the strike of the fault), S_Hmax_ swings toward the fault orientation, and similarly for S_hmin_ in a convergent setting (far-field S_hmin_ < 45° to the strike of the fault). For the special case where both S_Hmax_ and S_hmin_ are 45° to the fault then stress trajectories are unaffected. Shear stresses resisting movement along the Dead Sea fault are interpreted to be very low^62^, and the Ben-Avraham-Zoback model is probably applicable to the entire Gulf of Aqaba – Dead Sea transform segment.

**Figure 9:**
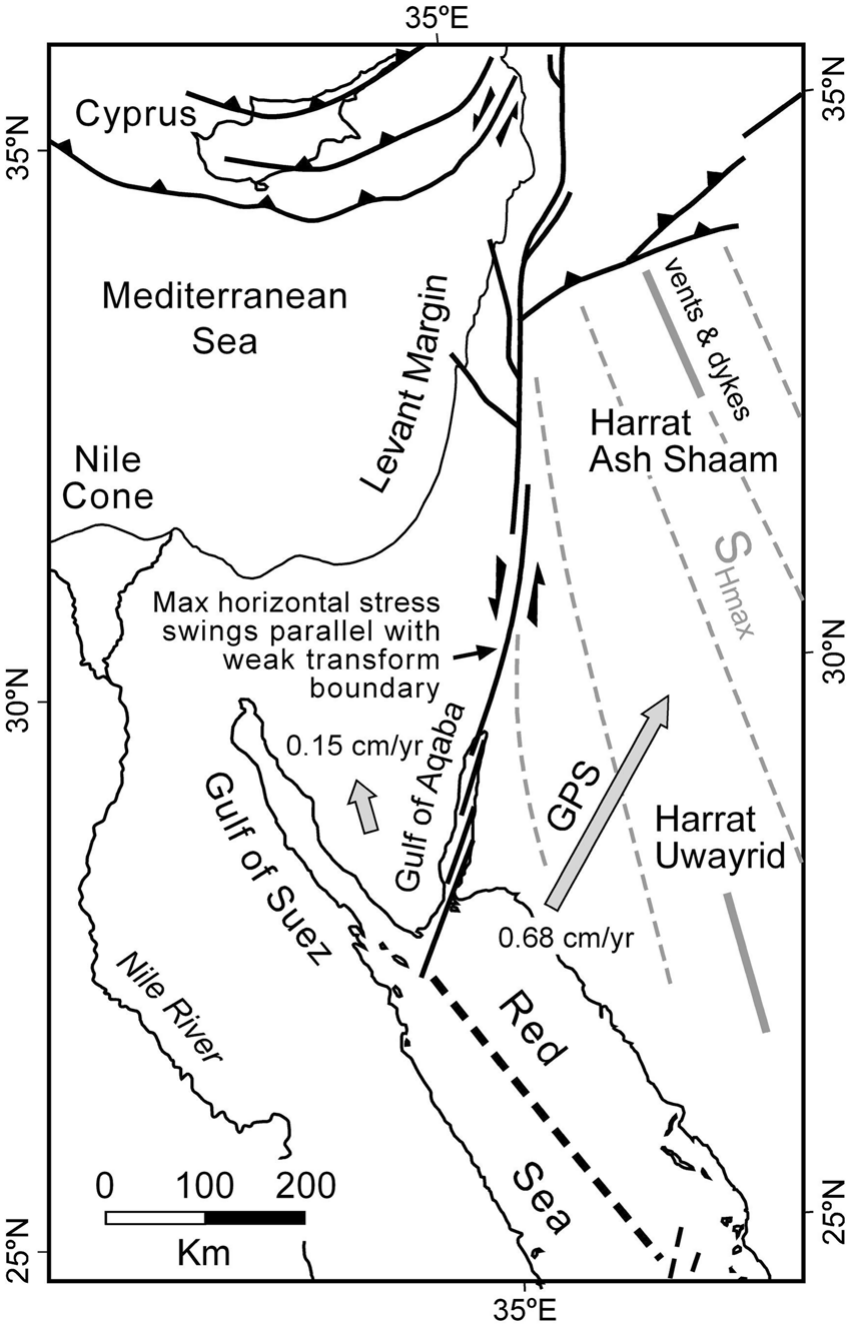
Evidence suggesting that the Gulf of Aqaba – Dead Sea transform plate boundary is partly divergent. Large arrows give GPS-determined plate motions of Sinai and Arabia relative to Africa/Nubia^7, 63^ and indicate a component of opening across the transform fault. Dashed grey lines are maximum horizontal stress trajectories (S_Hmax_) based on aligned vent and dike orientations (grey bars) at Harrats Ash Shaam and Uwayrid^64^. The trajectories are shown rotating into parallelism with the weak transform fault which places the minimum horizontal stress roughly perpendicular to the plate boundary. This allows both strike-slip and extensional faulting to occur simultaneously^25^. Figure was generated with Macromedia FreeHand MX v11.0.1 (https://www.adobe.com/mena_en/products/freehand/).

Two lines of evidence indicate that this is partly a divergent plate boundary and therefore should experience extensional faulting. GPS datasets suggest that Arabia is moving in a NNE direction relative to Africa/Nubia at ~0.68 cm/yr (Fig. 9)^7^. Sinai relative to Africa/Nubia is moving ~0.15 cm/yr toward the NNW^63^. These measurements confirm that there is a component of opening across the Gulf of Aqaba at a plate scale. We are unaware of present-day stress measurements on the Sinai micro-plate itself, but in western Arabia S_Hmax_ is aligned NNW making an angle of about 25–30° to the transform boundary (Fig. 9). This is based on observations of aligned Quaternary volcanic vents and dikes that are intruded parallel to S_Hmax_ at Harrats Ash Shaam and Uwayrid^64^. We suggest that the regional western Arabia S_Hmax_ trajectories rotate into parallelism with the transform boundary as proposed by Ben-Avraham and Zoback^25^ and facilitate the formation of transform-parallel extensional faults (Fig. 9). This then drives the footwall uplift that we have documented in Fig. 7.

The southward decrease in Gulf of Aqaba seismicity, and the associated decrease in footwall uplift, may reflect interaction between Gulf of Aqaba transform motion and northern Red Sea oblique incipient oceanic spreading^5^. If the northern Red Sea is attempting to propagate laterally into the Gulf of Aqaba then perhaps higher geothermal gradients are inhibiting brittle failure of the crust. However a recent tomographic inversion of regional earthquake data for the crust and uppermost mantle of the Gulf of Aqaba and northern Red Sea region complicates this scenario^68^. This study reported a prominent P and S high-velocity anomaly beneath the Red Sea from depths of 10 to 40 km. A more localized high-velocity anomaly occurs at 10 km depth beneath the Arnona-Aragonese Deep and the areas immediately east in Arabia. In the northern and southern Gulf of Aqaba the velocities are much slower. This implies that it is the shallow crust of the Arnona-Aragonese Deep that most closely resembles that of the northern Red Sea.

Why seismicity and footwall uplift decrease to the north in the Gulf of Aqaba is also not clear. The long-term slip rates in the northern Gulf of Aqaba and Arava Valley must match those to the north and south as there are no known intersecting strike-slip fault splays in this region. The earthquake recurrence interval for this section of the transform boundary may be much different than that of the central Gulf of Aqaba and perhaps our instrumental record is simply too short to adequately portray the true longer-term seismic picture.

The *M*_S_ 7.2 Nuweiba earthquake of 22 November 1995 is a clear example of the potential dangers associated with the active tectonics of the central Gulf of Aqaba. Our study suggests that the deformation processes occurring here are part of a long-term pattern and that therefore the future occurrence of similar or even larger events is highly probable. Human activity in this region, and in particular the construction of buildings, tunnels and bridges, should take this into consideration.

## Electronic supplementary material

Supplementary Information
