# Supplementary material for: Seismic Hazards Implications of Uplifted Pleistocene Coral Terraces in the Gulf of Aqaba
Source: Sci Rep. 2017 Feb 24;7:38. doi: 10.1038/s41598-017-00074-2 (PMC5428358; doi:10.1038/s41598-017-00074-2)
Supplement: Supplementary file 1 — Supplementary Information [file 41598_2017_74_MOESM1_ESM.pdf]

Supplementary Information

Seismic Hazards Implications of Uplifted Pleistocene Coral Terraces in the Gulf of Aqaba

W. Bosworth, P. Montagna, E. Pons-Branchu, N. Rasul, M. Taviani

U/Th analysis

Sixty-eight coral samples and two fragments of botryoidal aragonite were collected from 27 raised Pleistocene coral terraces sites for U-series dating. Among those samples, 32 coral fragments were selected for absolute chronology based on their appearance and the presence of well-preserved morphological features. In most cases, coral sub-samples were extracted from the internal part of massive *Porites* colonies or large *Acropora* branches. This portion often contains the most pristine coral fragments characterized by a fresh appearance resembling the aragonite skeleton of live-collected specimens. The “weathered” external portion was first removed in the field using a chisel and hammer. Further refinement of the samples for U-series dating was made in the lab using a small diamond cutting disc and a diamond bur. In particular, all the visible contaminations and sediment-filled cavities were removed and the samples were then crushed into small chunks (ca. 1-2 mm in size) with an agate and pestle. Finally, each sample was examined under a binocular microscope and any further contamination was removed by hand-picking. The botryoidal aragonite was also crushed into small chunks. An aliquot (ca. 10 mg) of carbonate of each sample was used for X-ray diffraction (XRD) analysis to identify the bulk mineralogy (see diffractometric analysis section). Seven samples with less than 2.5% of calcite were selected for U-series dating. The carbonate material of these samples was transferred to an acid cleaned Teflon beaker, ultra-sonicated in MilliQ water and leached with 0.1N HCl for 15 s. The samples were subsequently rinsed twice with MilliQ water and dried in an oven at 50°C overnight. Each sample (~ 60-80 mg) was completely dissolved in 3-4 ml dilute HCl (~10%) and mixed with an internal spike with known concentrations of ^{229}Th, ^{233}U and ^{236}U, calibrated against a Harwell Uraninite solution (HU-1) assumed to be at secular equilibrium. The

solutions were evaporated to dryness at 70°C, re-dissolved in 0.6 ml 3N HNO_3 and then loaded into 500 µl columns packed with UTEVA resin (Eichrom Technologies, USA) to isolate uranium and thorium from the other major and trace elements of the carbonate matrix. The U and Th separation and purification followed a procedure described in Pons-Branchu et al. (2014). The U and Th isotopes were measured using a Thermo Scientific™ Neptune^{Plus} MC-ICPMS installed at the Laboratoire des Sciences du Climat et de l'Environnement (Gif-sur-Yvette, France) following the analytical protocol reported in Pons-Branchu et al. (2014). The ^{230}Th/U ages were calculated from measured atomic ratios through iterative age estimation (Ludwig and Titterington, 1994), using the ^{230}Th, ^{234}U and ^{238}U decay constants of Cheng et al. (2013) and Jaffey et al. (1971). Due to the low ^{232}Th concentration (< 0.4 ng/g) and high ^{230}Th/^{232}Th ratio (>28,000) (see Table 2), no correction was applied for the non-radiogenic ^{230}Th fraction. The initial δ^{234}U ratio of the samples is higher than that of modern seawater (146.8‰; Andersen et al., 2010), indicating that they might have experienced open-system U-series behavior. This is likely caused by alpha recoil mobilization of ^{234}Th and ^{230}Th with simultaneous increase in the initial δ^{234}U value and ^{230}Th/^{238}U ratio and hence over-estimation of the age relative to a closed system (Thompson et al., 2003). However, five out of seven samples have initial δ^{234}U values that fall within the so-called “less strict” 8‰ range (table 2) and the ages can be considered as reasonable (Obert et al., 2016).

We calculated corrected U-series ages for open system using the open-system age equations of Thompson et al. (2003) (Table 2), even though our limited dataset does not allow us to confirm the expected mixing slopes predicted by the Thomson model. Overall, both conventional and corrected U-series ages fall within the time interval of the MIS5e last interglacial sea-level high-stand (e.g. Obert et al., 2016).

Petrographic observations and diffractometric analysis

Petrographic observations (optical microscope and SEM) and diffractometric measurements were carried out prior to geochemical analyses to check the quality of the samples. Petrographic observations enabled us to identify unaltered coral features such as centers of calcification (COCs)

and fibrous aragonite and to reveal any diagenetic modification. Diagenetic features such as early diagenetic submarine and freshwater vadose cements, diagenetic crystal growth and internal leaching were identified and, where possible, separated from the pristine material.

For the diffractometric analysis, the samples were crushed into a fine powder with a mortar and pestle and the powder was mounted on a silicon zero background sample holder. The X-ray powder diffraction data were recorded on a Panalytical θ-θ diffractometer at the Department of Geosciences of the University of Padova (Italy). The program High Score Plus was used for phase identification and quantitative analysis by Rietveld refinement (Rietveld, 1967). Refined parameters were scale factors, zero-shift, background, lattice constants, and profile parameters (Gaussian and Lorentzian coefficients).

References

Andersen, M. B., Stirling, C. H., Zimmermann, B., Halliday, A. N. Precise determination of the open $^{234}U/^{238}U$ composition. Geochemistry, Geophysics, *Geosystems* **11**, Q12003, doi:10.1029/2010GC003318 (2010).

Cheng, H. et al. Improvements in ^{230}Th dating, ^{230}Th and ^{234}U half-life values, and U/Th isotopic measurements by multi-collector inductively coupled plasma mass spectrometry. *Earth Planet. Sci. Lett*. **371-372**, 82–91 (2013).

Jaffey, A. H., Flynn, K. F., Glendenin, L. E., Bentley, W. C., Essling, A. M. Precision measurement of half-lives and specific activities of ^{235}U and ^{238}U. *Phys. Rev. C 4* **5**, 1889–1906 (1971).

Obert J. C. et al. $^{230}Th/U$ dating of Last Interglacial brain corals from Bonaire (southern Caribbean) using bulk and theca wall material. *Geochimica et Cosmochimica Acta* **178**, 20-40 (2016).

Pons-Branchu, E. et al. A geochemical perspective on Parisian urban history based on U-Th dating, laminae counting and yttrium and REE concentrations of recent carbonates in underground aqueducts. *Quat. Geochron.* **24**, 44-58 (2014).

Rietveld, H. M.. Line profiles of neutron powder-diffraction peaks for structure refinement. *Acta Crystallographica* **22**, 151-152 (1967).

Thomson, W. G., Spiegelman, M. W., Goldstein, S. L., Speed, R. C.. An open-system model for U-series age determinations of fossil corals. *Earth Planet. Sci. Lett.* **210**, 365-381 (2003).
